# Validating the reproducibility of a low-cost single-channel fNIRS device across hierarchical cognitive tasks

**DOI:** 10.3389/fnins.2024.1351341

**Published:** 2024-04-24

**Authors:** Shiyang Xu, XingLing Zeng, Fuxian Yin, Chao Zhang

**Affiliations:** ^1^Faculty of Education Science, Shanxi Normal University, Taiyuan, China; ^2^Faculty of Health Sciences, University of Macau, Macau, Macao SAR, China; ^3^Centre for Brain and Cognitive Sciences, University of Macau, Macau, Macao SAR, China; ^4^Faculty of Medicine, Macau University of Science and Technology, Macau, Macao SAR, China

**Keywords:** fNIRS, cognitive tasks, low-cost fNIRS device, prefrontal cortex activation, open-source hardware

## Abstract

This study evaluates a low-cost, single-channel fNIRS device in cognitive neuroscience, aiming to overcome the financial barriers of commercial systems by testing its efficacy in tasks of varying complexity. Twenty-six participants engaged in motor control (finger-tapping), working memory (n-back), and creativity (AUT) tasks while their prefrontal cortex activity was monitored using the device, with behavioral and cerebral blood flow changes recorded. Results showed the device’s capability to detect significant blood flow variations across different tasks, thereby supporting its use in cognitive research. The study confirms the potential of single-channel fNIRS as a cost-effective tool for diverse cognitive assessments, from simple motor actions to complex creative thinking.

## Introduction

In the realm of cognitive neuroscience, functional near-infrared spectroscopy (fNIRS) has emerged as a key tool for non-invasively probing the hemodynamic responses that accompany neural activity. This technology allows researchers to observe brain activity through the skull, facilitating the study of cognitive functions and neurodevelopmental processes (Boas et al., 2014). Despite the significant potential of fNIRS, its integration into broader research practices has been impeded by the high cost of commercial systems, which has limited its accessibility to primarily well-funded laboratories (Pinti et al., 2018). This restricted availability has posed challenges for the validation and reproducibility of data, hindering the engagement of a wider demographic with fNIRS technologies. Consequently, it becomes difficult to extend research findings to these populations for validation.

In response to these challenges, the field has seen the emergence of single-channel fNIRS systems using a do-it-yourself (DIY) or low-cost approach (Bracken et al., 2019; Boere et al., 2024). Yet, the current literature indicates a need for more systematic and comprehensive investigations into these single-channel fNIRS systems. While there is evidence of their potential in cognitive tasks, an exploration across a broader range of cognitive functions is lacking. Furthermore, the emphasis on the DIY aspect has often overshadowed the necessity for rigorous validation against established cognitive benchmarks (Arivudaiyanambi et al., 2020; Hag et al., 2021).

The integration of fNIRS into everyday neuroscience research and its application across various cognitive tasks have been highlighted as the next frontier in expanding the technology’s reach and impact (von Lühmann et al., 2021; Yücel et al., 2021; Liu et al., 2022). Recent advancements in mobile fNIRS technology present promising avenues for neurofeedback training and hyperscanning applications, as discussed in Konrad et al. (2024) and Klein et al. (2023). These developments are pivotal as they facilitate the deployment of fNIRS in dynamic, real-world settings, extending its utility beyond conventional laboratory environments. These pioneering efforts underscore the importance of developing versatile and cost-effective fNIRS systems that maintain methodological rigor while being accessible to a broader base of researchers and practitioners.

This study introduces a single-channel fNIRS device, designed to capitalize on the financial and operational benefits inherent to this technology. Notably, single-channel fNIRS systems are markedly more affordable than their multi-channel counterparts, enabling a broader spectrum of researchers, particularly those in under-resourced settings, to undertake cognitive neuroscience research. The simplicity and portability of these devices, coupled with their capacity for focused measurements, make them well-suited for field studies and for targeting specific cortical regions in hypothesis-driven research. Their ease of use further extends their utility to a diverse user base, including clinicians and educators without extensive training in neuroimaging. Although multi-channel systems provide detailed cerebral activity mapping, the single-channel fNIRS device presented herein is a valuable tool for focused cognitive investigations, offering a pragmatic and user-friendly option for diverse research applications.

The current study represents a significant advancement in the application of fNIRS technology. While previous research has demonstrated the feasibility of using fNIRS for basic motor tasks and simple cognitive exercises, this study extends this validation to encompass a more comprehensive range of cognitive functions, from primary motor actions to complex cognitive tasks.

Our research breaks new ground by conducting a comprehensive evaluation of a single-channel fNIRS device across a range of cognitive tasks, highlighting its versatility in both laboratory and real-world settings. We have rigorously tested the device on tasks demanding high cognitive functions like working memory and creativity. The results demonstrate the device’s capability to detect neural activity during complex tasks, suggesting its practicality for applications in clinical research, cognitive neuroscience, and education. This work lays the groundwork for future studies, paving the way for advancements in portable neuroimaging technology and its applications.

Our study aims to bridge these gaps by deploying a low-cost, modified single-channel fNIRS device in a battery of cognitive tasks that span different levels of complexity. We have designed this study to assess cognitive abilities through a spectrum of tasks, including the finger-tapping task for motor control, n-back tasks for working memory, and the Alternate Uses Test (AUT) for creativity assessment (Hag et al., 2021; Rocco et al., 2021; Beaty et al., 2022; Qiao et al., 2022; Figeys et al., 2023).

We hypothesize that despite the modest cost of the modified fNIRS device, it will prove to be a reliable and valid tool for assessing cognitive functions across these tasks. Confirming this hypothesis would represent a significant step forward in cognitive neuroscience by providing a cost-effective and replicable instrument for research, thereby democratizing the application of fNIRS technology in cognitive studies (Xiong et al., 2014; Rieiro et al., 2019; Ren et al., 2022; Gao et al., 2023).

## Method

### Participants

The study involved 26 participants (10 males, 16 females; *M* = 20.58 years, SD = 2.78). All participants were free from psychiatric disorders and had normal or corrected-to-normal vision. Prior to the experiment, each participant provided informed consent. The experiment was approved by the Ethics Committee of Shanxi Normal University. Due to one participant withdrawing from the Alternate Uses Task (AUT), the total number of participants for that task was 25.

### Procedure

The experiment was conducted on a computer system equipped with PsychoPy software (Peirce et al., 2019). Participant responses were monitored and recorded using a standard keyboard. An additional setup facilitated the broadcasting of event markers through the Lab Streaming Layer (LSL) for real-time data streaming.

#### Finger-tapping task

Participants were instructed to engage in a preparatory 15-s rest period, symbolized by a ‘+,’ prior to the commencement of the motor task. Subsequently, they carried out a finger-tapping task, responding to visual cues by tapping the corresponding key. The experimental setup included a standard keyboard connected to a computer. Participants positioned their fingers on the specified keys, with the right index finger on ‘J,’ and the remaining fingers on ‘K,’ ‘L,’ and ‘;’ keys.

The task was divided into 5 blocks, each comprising a series of trials. In each trial, a cue was presented, followed by the participant’s response. The structure of the experiment consisted of multiple blocks, each separated by a rest period. Each trial had a duration of 30 s, alternated with 15-s rest intervals and 15-s periods of active tapping. The tapping frequency and accuracy were measured and recorded.

#### N-back task

Participants were instructed to observe a sequence of colored rectangles and identify if they had seen the same color in a specified number of previous one or two trials. The experiment featured two rounds with distinct instructions. In the first round (1-back), participants pressed the ‘F’ key for color matches with the previous rectangle and the ‘J’ key for non-matches. The second round (2-back) involved comparing the current rectangle with the one from two trials prior. Instructions mirrored the first round, with ‘F’ for matches and ‘J’ for non-matches. Each round began with detailed instructions to ensure comprehension. There are 6 discrete blocks each for the 1-back and 2-back tasks, wherein each block comprises a 16-s rest period followed by 20-s n-back task trials.

Each trial presented a fixation cross (‘+’) briefly, followed by a colored rectangle. The sequence of rectangles was randomized for each participant. Rectangles appeared for 2 s each, with a 0.3-s inter-stimulus interval marked by the fixation cross. Participant responses, reaction times, and accuracy were recorded.

#### Alternate uses task

In the AUT, participants viewed an stimuli word (‘brick’) and generated the answer as many uses as possible, measuring divergent thinking and creativity. The task was self-paced, with participants pressing the space bar upon completing their list for each item. A 13 s rest periods were indicated by a fixation cross on the screen.

### Statistical analysis

The behavioral data were analyzed using JASP software (JASP Team, 2023). Descriptive statistics were computed to obtain the mean and standard deviation of reaction time and accuracy for the finger-tapping and n-back tasks across conditions. A paired-samples t-test was conducted to examine the difference between the 1-back and 2-back conditions in the n-back task. The effect size was calculated using Cohen’s d. For the AUT, the mean number of responses per item was calculated as a measure of originality, flexibility, and fluency scores. The main results and the significance level (*p* < 0.05) were reported.

### Hardware

**Figure 1:**
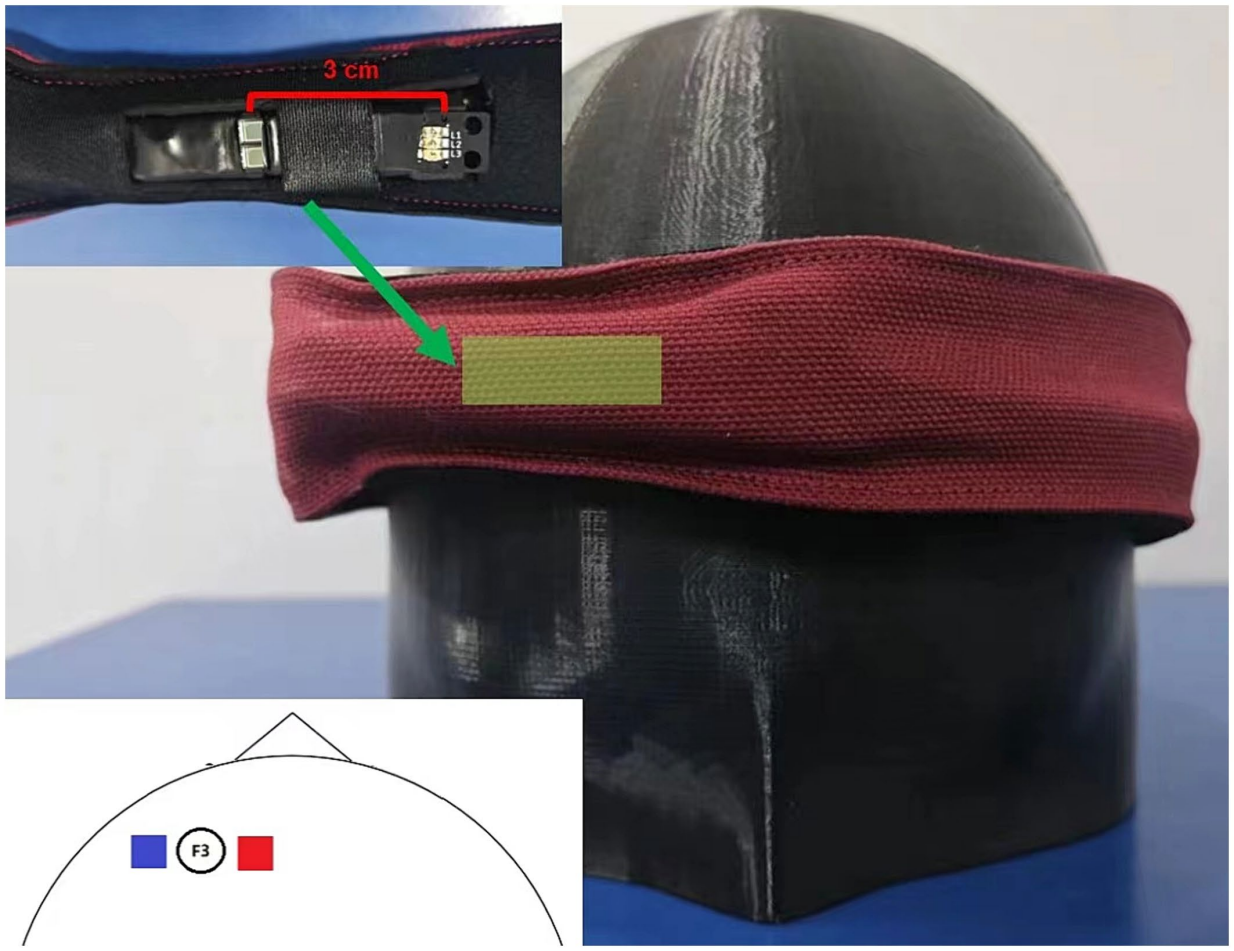
Hardware setup. This figure illustrates the hardware configuration of the modified HEGduino fNIRS device. The inset shows a close-up view of the sensor unit, which includes a photodetector (PD) and a light-emitting diode (LED). The main image depicts the device as worn on the head, over the left dorsolateral prefrontal cortex, with the sensor band in place.

The HEGduino V2 device (Joshbrew, 2023) was strategically equipped with LEDs that emit at specific wavelengths suitable for capturing hemodynamic changes in the brain (Figure 1). Initially, the device included LEDs at 660 nm, 880 nm, and 940 nm wavelengths. However, a minor firmware update led us to replace the 940 nm LED with one that emits at 880 nm, to better match the absorption peaks of hemoglobin and thus refine the sensitivity of the device.

In alignment with the open-source ethos demonstrated by Tsow et al. (2021), we have provided a comprehensive breakdown of the hardware components used in our modified fNIRS device. This information is designed to assist interested researchers in replicating our DIY setup for their own studies. The detailed list of components, along with the specifications and modifications made, are available in the Supplementary material.

In alignment with the open-source ethos demonstrated by Tsow et al. (2021), we have provided a comprehensive breakdown of the hardware components used in our modified fNIRS device. This information is designed to assist interested researchers in replicating the setup for their own studies. The detailed list of components, along with the specifications and modifications made, are available in the Supplementary material.

### Optode placement

Our single-channel fNIRS optode was positioned to monitor activity in the dorsolateral prefrontal cortex (dlPFC). The dlPFC is associated with executive functions and working memory and is a region of considerable interest in cognitive neuroscience research (Fitzgerald et al., 2009). To accurately target the dlPFC, we placed our optode at the F3 location, which corresponds to the left dlPFC (Figure 1).

### Software and data analysis

The in-house software developed for this study is integral to the data acquisition and analysis process for our fNIRS measurements. It includes a series of signal processing algorithms, artifact detection and correction protocols, and a graphical user interface (GUI) for real-time monitoring and analysis. For further details regarding the software used in this study, please see the Supplementary materials. The custom software developed for our study custom-designed software and representative data used for evaluation can be accessed at https://osf.io/yrwaf/.

### fNIRS data analysis

#### Conversion to optical density

The raw intensity data captured from the fNIRS device were first transformed into optical density (OD) measurements. This initial step is crucial as it accounts for variations in light intensity due to the different path lengths of light through the tissue, adhering to the modified Beer–Lambert Law.

#### Motion artifact detection

Motion artifacts were detected by identifying segments of data that exhibit abrupt changes indicative of motion-related artifacts. The parameters for detection were set to identify motion over a 0.2-s window and mark data over ±0.1 s around the identified motion artifact. A threshold multiplier of 10 and an amplitude threshold of 0.3 were used for precise artifact detection.

#### Motion artifact correction

Upon detection, motion artifacts were corrected by applying a spline interpolation method to the OD data. This correction method is based on fitting splines to the data to approximate the signal during periods of motion, replacing the artifact-affected segments. The spline tension parameter was set to 0.99 to achieve a balance between fit and smoothness.

#### Bandpass filtering

The motion-corrected OD data were then subjected to bandpass filtering to reduce the influence of high-frequency noise and slow drifts that are not related to cerebral hemodynamics. The filter was set with a low cutoff frequency of 0.01 Hz and a high cutoff frequency of 0.1 Hz, which are typical for capturing the hemodynamic response.

### Signal-to-noise ratio assessment

In response to the insightful suggestion from the reviewer, we have incorporated an evaluation of the signal-to-noise ratio (SNR) for our study. The SNR assessment provides a quantitative measure of the quality of our measurements, and it is crucial for validating the efficacy of our modified HEGduino device across different cognitive tasks.

We calculated the SNR for three distinct tasks using the methodology proposed by Boere et al. (2024). The tasks analyzed were the Finger Tapping Task (FGT), the Alternate Uses Task (AUT), and the N-back Task, which are representative of motor, creative, and working memory functions, respectively.

## Results

### Behavior results

#### Finger-tapping task

The finger-tapping task revealed an average accuracy rate of 0.68 with a mean response time of 0.62 s, indicating moderate performance accuracy and relatively quick motor responses.

#### N-back task

Statistical analysis of the n-back task via paired-samples *t*-tests revealed significant differences in both the accuracy and response times between the 1-back and 2-back conditions.

Participants were more accurate in the 1-back condition (*M* = 0.859, SD = 0.156) compared to the 2-back condition (*M* = 0.659, SD = 0.125), *t*(25) = 5.477, *p* < 0.001, with a large effect size (Cohen’s *d* = 1.074, SE = 0.324). Moreover, participants responded faster in the 1-back condition (*M* = 0.659, SD = 0.114) than in the 2-back condition (*M* = 0.986, SD = 0.154), *t*(25) = −9.020, *p* < 0.001, also with a large effect size (Cohen’s *d* = −1.769).

#### Alternate uses task

For the AUT task, participants demonstrated an average Originality score of 9.5 (SD = 4.12), Flexibility score of 6.19 (SD = 3.21), and a Fluency score of 5.62 (SD = 3.18). These scores indicate the participants’ ability to generate a moderate number of novel and varied uses for a common object.

### fNIRS results

**Figure 2:**
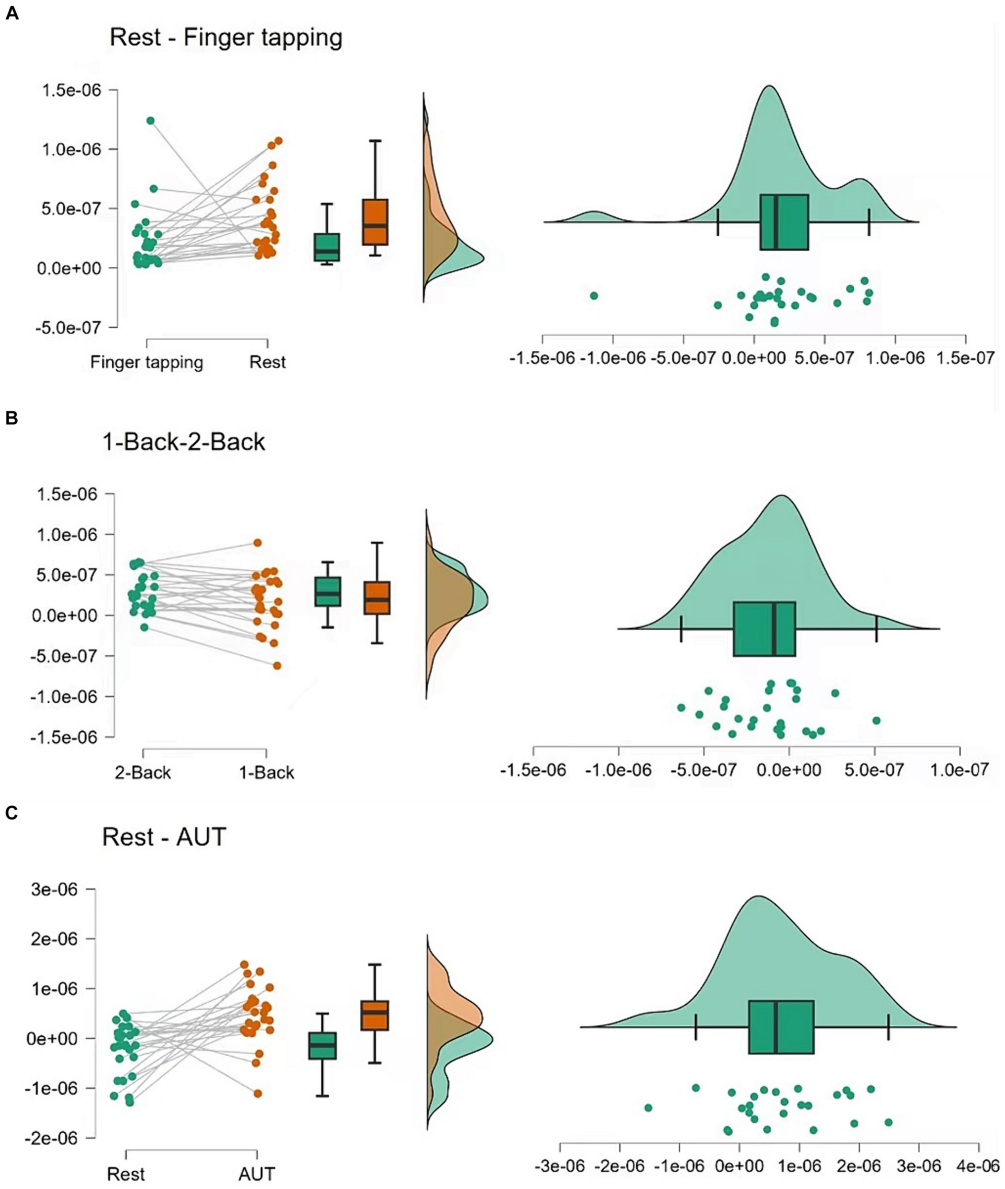
Hemodynamic responses during tasks. **(A)** Displays the hemodynamic response, indicated by changes in oxyhemoglobin (ΔHbO), during a finger-tapping task. **(B,C)** Show the ΔHbO during the n-back tasks (1-back and 2-back) and the Alternate Uses Test (AUT), respectively. These curves represent the averaged responses, time-locked to the onset of the tasks.

**Figure 3:**
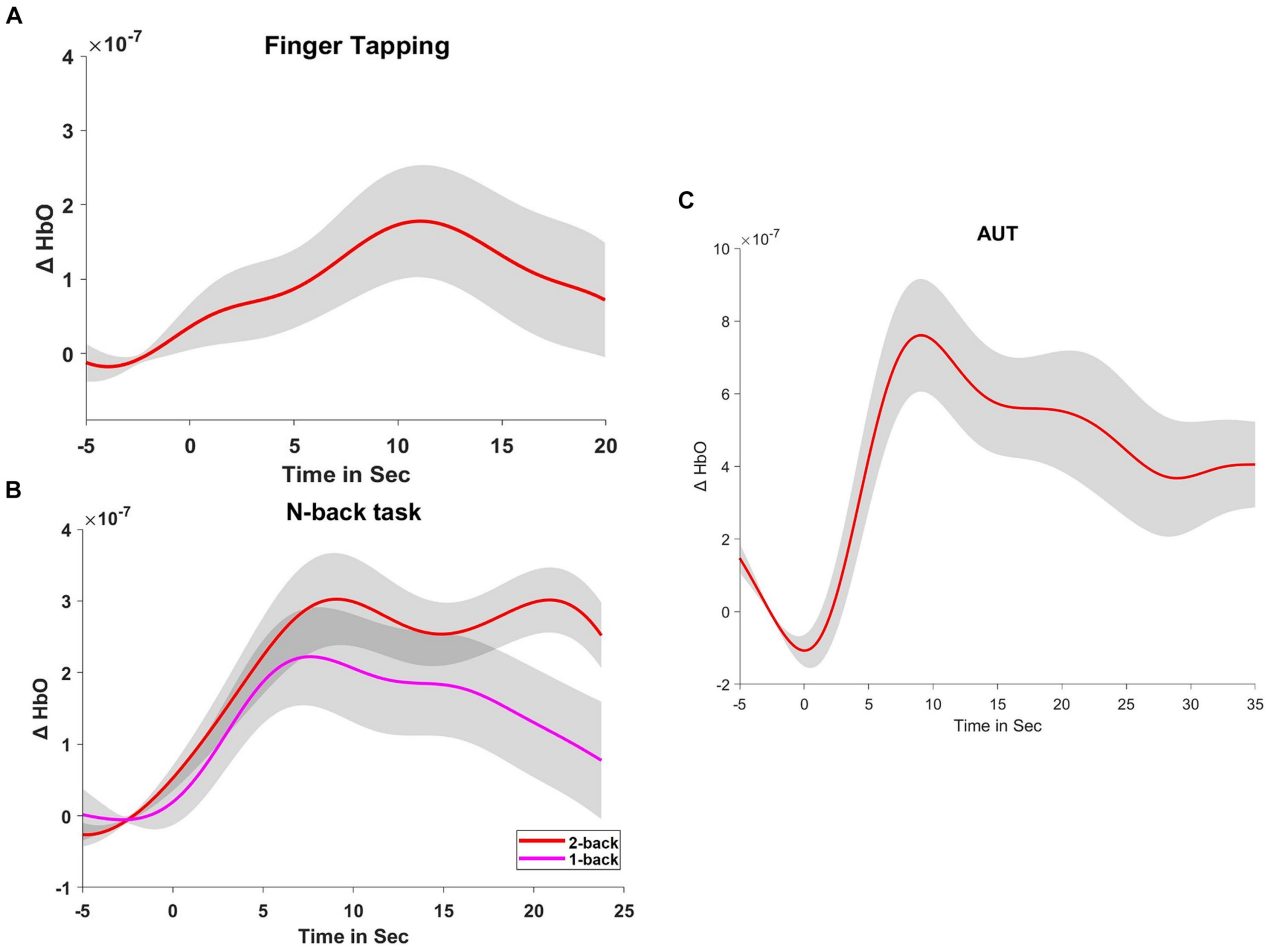
Statistical analysis of hemodynamic responses. **(A)** Compares the changes in ΔHbO between the 1-back and 2-back conditions, showcasing individual participant data and summary statistics. **(B)** Contrasts the rest and finger-tapping task conditions, and **(C)** contrasts the rest and AUT task conditions, both including raw data points, boxplots, and density plots, illustrating the distribution of responses and central tendency.

The results from the behavioral data and the fNIRS measurements suggest that both cognitive and motor tasks actively engage the dorsolateral prefrontal cortex, with varying degrees of neural activation depending on the task complexity (Figures 2, 3).

#### Finger-tapping task

The fNIRS data for the finger-tapping task compared to the resting state indicated a significant difference in cerebral blood flow, *t*(25) = 2.484, *p* = 0.020, with a moderate effect size (Cohen’s *d* = 0.487). This change reflects heightened neural activity in the left dorsolateral prefrontal cortex during the motor task.

#### N-back task

Comparison of the cerebral blood flow of dlPFC between the 1-back and 2-back conditions revealed a significant difference, *t*(25) = −2.341, *p* = 0.028, with a moderate effect size (Cohen’s *d* = −0.459). The 1-back condition (*M* = 1.711 × 10^−7^, SD = 3.328×10^−7^) showed slightly higher cerebral blood flow than the 2-back condition (*M* = 2.923×10^−7^, SD = 2.289×10^−7^), suggesting increased neural engagement during the less complex 1-back task.

#### AUT task

During the resting state compared to the AUT task, fNIRS data showed a significant increase in cerebral blood flow, *t*(24) = 3.641, *p* < 0.001, with a large effect size (Cohen’s *d* = 0.728). This suggests that the AUT task was associated with increased cerebral blood flow in the left dorsolateral prefrontal cortex, indicating greater neural activation.

### Signal-to-noise ratio assessment

#### Finger tapping task

The mean SNR was found to be 30.307. This high SNR indicates a strong signal fidelity during the motor task.

#### Alternate uses task

For the creativity task, the mean SNR was 23.883, suggesting reliable signal detection during cognitive processes involving divergent thinking.

#### N-back task

During the working memory task, the mean SNR was 6.302. Although lower in comparison to the other tasks, this SNR still indicates an acceptable level of signal quality for cognitive load assessments.

## Discussion

The present study investigated the reliability and validity of a low-cost, single-channel functional near-infrared spectroscopy (fNIRS) device across a range of cognitive tasks, including motor control, working memory, and creative thinking. The findings support the use of this economical fNIRS system in cognitive neuroscience research, demonstrating its potential to measure cerebral blood flow changes corresponding to different cognitive processes.

The results revealed significant differences in prefrontal cortex activation across the task complexity gradient, particularly between the 1-back and 2-back working memory tasks. This differentiation in hemodynamic response is consistent with previous research using commercial fNIRS systems, indicating that increased cognitive load results in heightened prefrontal cortex activity (Figeys et al., 2023). Furthermore, the observed correlation between behavioral performance on cognitive tasks and fNIRS data aligns with existing literature, reinforcing the device’s validity in cognitive task assessment (Bracken et al., 2019; Saikia et al., 2021; Boere et al., 2024).

Importantly, the study extends the utility of low-cost fNIRS devices beyond merely demonstrating technical feasibility, as seen in prior DIY fNIRS device or EEG headset (Bracken et al., 2019; Rieiro et al., 2019; Boere et al., 2024). By systematically evaluating the device across multiple cognitive domains, we provide empirical evidence for its robustness and versatility as a research tool. This is particularly crucial given the skepticism that often surrounds the reliability of low-cost medical or research equipment.

The implications of this research are manifold. For one, the validation of a low-cost fNIRS device opens the possibility for its use in a wider range of settings, including educational and clinical environments where funding for high-end equipment may not be available (Rieiro et al., 2019; Tsow et al., 2021). Moreover, the potential for this technology to be used in neurofeedback training could enhance cognitive performance or rehabilitation programs, echoing findings from related EEG studies (Ortuño-Miró et al., 2023). The affordability of low-cost fNIRS devices also paves the way for improved multi-person interactions and cost-efficient educational applications (Wang et al., 2018; Chen et al., 2023).

Our results have shown a consistency with previous studies that have utilized multi-channel fNIRS systems in terms of the overall patterns of hemodynamic responses during cognitive tasks. For instance, our findings in the finger-tapping and n-back tasks align with established literature indicating increased prefrontal cortex activity, reflecting the engagement of motor and working memory processes (Brigadoi et al., 2012; Fishburn et al., 2014; Herff et al., 2014; Sommer et al., 2021).

This research has its own set of constraints, yet it also opens avenues for future directions. In this pilot study, we recognize that the limited number of participants presents a challenge in conducting a robust test–retest reliability assessment. Despite this constraint, our pilot study provides valuable insights into the potential applications of a low-cost, open-source fNIRS system. Then, the lack of short-distance channels in our current device configuration may lead to an overestimation of the fNIRS signals attributed to brain activity, as physiological fluctuations such as heartbeat, respiration, and Mayer waves can contribute to the measured signals. Finally, while HbO provides valuable insights into cerebral oxygenation, the absence of deoxyhemoglobin (HbR) measurements presents a limitation, as it offers complementary information on cerebral blood volume and oxygen extraction. The current limitation in reporting HbR is due to technical constraints; however, we are actively working to address this in our forthcoming research, as detailed in Konrad et al. (2024). Acknowledging the intricacies presented by this pilot study, we advocate for the adoption of preregistration in subsequent research endeavors. This approach, as championed by Schroeder et al. (2023), will enhance the clarity and reproducibility of our scientific inquiry, allowing the researcher to systematically address biases and substantiate the reliability of our results.

Future research should aim to replicate these findings across diverse populations and investigate the long-term reliability of the device over repeated use. The integration of low-cost fNIRS with other neuroimaging methods, such as EEG, could provide a more detailed picture of the neural substrates underlying cognitive functions (Rybář, 2023). Further, exploring the applicability of this device in clinical populations and for neurofeedback interventions would broaden its potential impact. Future research and development efforts will be directed toward incorporating short-distance channels into our device design, which will allow for more accurate measurements and interpretations of fNIRS data.

## Conclusion

In conclusion, the low-cost single-channel fNIRS device has shown promise as a possible tool for cognitive neuroscience research. It offers a practical and cost-effective alternative to commercial systems, potentially democratizing access to neuroimaging technology and facilitating the expansion of cognitive research into new domains and applications. The observed consistency with previous multi-channel fNIRS studies and the strong correlation between behavioral performance and hemodynamic responses underscore the device’s credibility.

As we look to the future, we anticipate that low-cost fNIRS technology will play an increasingly important role in cognitive neuroscience, enabling researchers to investigate brain function in more naturalistic settings and to explore the neural underpinnings of complex social interactions. With continued refinement and validation, devices like the one presented in this study have the potential to revolutionize our understanding of the human brain and behavior.

## Data availability statement

The raw data supporting the conclusions of this article will be made available by the authors, without undue reservation.

## Ethics statement

The studies involving humans were approved by Faculty of Educational Sciences, Shanxi Normal University. The studies were conducted in accordance with the local legislation and institutional requirements. The participants provided their written informed consent to participate in this study.

## Author contributions

SX: Conceptualization, Writing – original draft. XZ: Conceptualization, Methodology, Software, Writing – review & editing. FY: Conceptualization, Writing – review & editing. CZ: Supervision, Writing – original draft, Writing – review & editing.
