# Supplementary material for: Validating the reproducibility of a low-cost single-channel fNIRS device across hierarchical cognitive tasks
Source: Front Neurosci. 2024 Apr 24;18:1351341. doi: 10.3389/fnins.2024.1351341 (PMC11076663; doi:10.3389/fnins.2024.1351341)
Supplement: Supplementary file 1 [file Table_1.DOCX]

**Supplementary Material 1 - Detailed Hardware Information**

**Firmware and LED Specification**

The HEGduino V2 firmware has been meticulously fine-tuned to enhance its performance for fNIRS applications. We replaced standard LEDs with high-intensity, narrow-spectrum LEDs at 660nm and 880nm wavelengths. The 660nm LED, commonly utilized in pulse oximetry, operates within the red-light spectrum and is chosen for its strong absorption by deoxygenated hemoglobin. The 880nm LED lies within the near-infrared spectrum, which has been selected for its deeper tissue penetration capacity, crucial for monitoring cerebral blood flow and for its peak absorption by oxygenated hemoglobin. These modifications aim to boost the device's sensitivity and specificity in detecting fluctuations in cerebral oxygenation and hemoglobin deoxygenation levels.

**Design Accessibility**

Affordably priced at just US$80, this ensemble stands as a cost-effective solution relative to its commercial counterparts. The entire kit, comprising the ESP32 microcontroller and a simple yet robust arrangement of three LEDs and two photodiodes, offers a frugal but powerful option for fNIRS research.

The PCB design files and the bill of materials (BOM) are hosted on GitHub (https://github.com/joshbrew/HEG_ESP32_Delobotomizer), ensuring transparency and facilitating replication and innovation.

We acknowledge the significant contributions of Joshua Brewster, whose innovative design of the HEGduino V2 hardware has been shared under an open-source license. His work has effectively reduced the barriers to conducting fNIRS research, making it more attainable for a wider scientific community. The device's availability on Crowd Supply exemplifies a shared ethos of community support and engagement within the

**Key features of the software include:**

**Signal Preprocessing:** The software performs noise filtering, signal normalization, and temporal smoothing to prepare raw fNIRS data for analysis.

**Artifact Rejection:** Utilizing both automated and manual checks, the software identifies and corrects for motion artifacts and physiological noise, ensuring data quality.

**Data Visualization:** Researchers can observe hemodynamic responses in real-time through the GUI, which displays oxygenated and deoxygenated hemoglobin concentration changes.

**Supplementary Material 2 - AUT Ratings and Rater Qualifications**

For the statistical analysis of the AUT, we employed a panel of raters who assessed the participants' responses based on three dimensions: fluency, originality, and flexibility. The raters were trained and supervised by an expert teacher of creativity with extensive experience in evaluating cognitive tasks that measure creative thinking.

**Supplementary Material 3- Intraclass Correlation Coefficient (ICC) Results**

We have calculated the intraclass correlation coefficient (ICC) for a typical task. The ICC is a measure of test-retest reliability, which assesses the consistency of measurements across different time points or raters.

For the n-back task the ICC for the n-back task was found to be 0.573, indicating moderate reliability. We acknowledge the limitations of providing an ICC estimate from such a small sample. Future studies with larger sample sizes are needed to obtain more stable and reliable ICC estimates.
